# DNA duplication is essential for the repair of gastrointestinal perforation in the insect midgut

**DOI:** 10.1038/srep19142

**Published:** 2016-01-12

**Authors:** Wuren Huang, Jie Zhang, Bing Yang, Brenda T. Beerntsen, Hongsheng Song, Erjun Ling

**Affiliations:** 1 Environment and Plant Protection Institute, Chinese Academy of Tropical Agricultural Sciences, Haikou, Hainan 571101, China; 2 Key Laboratory of Insect Developmental and Evolutionary Biology, Institute of Plant Physiology and Ecology, Shanghai Institutes for Biological Sciences, Chinese Academy of Sciences, Shanghai 200032, China; 3 Veterinary Pathobiology, University of Missouri, Columbia, MO 65211, USA; 4 School of Life Sciences, Shanghai University, Shanghai 200444, China

## Abstract

Invertebrate animals have the capacity of repairing wounds in the skin and gut via different mechanisms. Gastrointestinal perforation, a hole in the human gastrointestinal system, is a serious condition, and surgery is necessary to repair the perforation to prevent an abdominal abscess or sepsis. Here we report the repair of gastrointestinal perforation made by a needle-puncture wound in the silkworm larval midgut. Following insect gut perforation, only a weak immune response was observed because the growth of *Escherichia coli* alone was partially inhibited by plasma collected at 6 h after needle puncture of the larval midgut. However, circulating hemocytes did aggregate over the needle-puncture wound to form a scab. While, cell division and apoptosis were not observed at the wound site, the needle puncture significantly enhanced DNA duplication in cells surrounding the wound, which was essential to repair the midgut perforation. Due to the repair capacity and limited immune response caused by needle puncture to the midgut, this approach was successfully used for the injection of small compounds (ethanol in this study) into the insect midgut. Consequently, this needle-puncture wounding of the insect gut can be developed for screening compounds for use as gut chemotherapeutics in the future.

The integument is the first line to protect insects from pathogen infection^1^,^2^. Epidermal cells of the integument express many proteins to form thick cuticles that are solid enough to protect insects^3^. During ecdysis, the molting fluids that accumulate between the old and new cuticle have protective functions^4^,^5^. However, in the environment, insect integuments are sometimes damaged by physical injuries, parasitoids and pathogens^6^,^7^. A wound in the insect integument can lead to bleeding, and then many pathogens can easily enter the wound. Thus, a wound in the insect integument must be repaired quickly. During the process of wound healing, hemolymph clots and melanization occurs to form a plug in the wound gap and then a scab forms over the wound, which serves to protect the insect from excessive bleeding and subsequent pathogen infection^8^. To induce clotting, several important proteins like transglutaminase and prophenoloxidase (PPO) are necessary to crosslink hemolymph and cuticle proteins surrounding the wound site^9^,^10^. When two PPO genes were deleted in *Drosophila melanogaster*, the hemolymph did not readily clot, and bleeding could not be stopped in time to prevent fly death^9^, which corroborates that insect PPO contributes to wound repair^8^. In addition, the aggregation of hemocytes around wound sites is advantageous for repair^11^. When an integument wound was repaired, the epidermal cells did not proliferate in *Drosophila*^8^,^12^. Instead, the cells near the integument wound oriented toward it and fused to form a syncytium, which is independent of scab formation and Jun N-terminal kinase (JNK) pathway activity^8^. Subsequently, via bsk (basket) and JNK pathway activity, the epidermal cells spread out and were re-epithelialized around the wound to aid in repair^8^. Consequently, wound repair in the insect integument involves a number of factors.

Wound repair in the insect midgut is different than what is observed for the insect integument. The insect gut is the largest organ and is used for food digestion and nutrient absorption^13^,^14^. There are likely many microorganisms in and on insect food. Recent work shows that there are PPOs in the foreguts and hindguts of many insects^15^,^16^. PPOs are secreted into the foregut contents where POs detoxify plant phenolics in the diet^16^, but may not be able to clean food bacteria. Wounds can occasionally form in the insect gut following ingestion of food and other items contaminated with detergent^17^, paraquat^18^,^19^ or toxins secreted by pathogens^20^,^21^. Using a transgenic approach, apoptosis was induced in specific cells of *Drosophila* midguts^12^,^15^. Unlike integument wound repair, the JAK/STAT and EGFR pathways were activated following the release of the ligands Upd3, vein and keren, which induced the intestinal stem cells to divide and differentiate leading to wound repair^21^,^22^. Stem cell division is very important for midgut wound repair in *Drosophila*^22^, which is different from the mechanism of wound repair in the *Drosophila* integument. Consequently, as a model, the studies described above offer important information to understand wound repair and regeneration in humans^23^.

Gastrointestinal perforation, which is a hole in the human gastrointestinal system, is a serious condition because the contents of the intestines can leak into the abdominal cavity^24^. In such a case, surgery is necessary to repair the perforation to prevent an abdominal abscess or sepsis^24^. In insects what happens when a physical perforation is made to the midgut? Can the insect be infected like humans? Is it possible for the midgut to repair itself? Thus, a study on the repair of a physical wound in the insect midgut is necessary, which may be helpful to understand the gastrointestinal perforation in humans.

In this study, we used a sterile needle to puncture the silkworm larval midgut through the integument which is similar to a gastrointestinal perforation in humans. Surprisingly, larvae that received a needle puncture did not die. Because there was not widespread antimicrobial peptide production noted, this result suggested that there was no systemic infection present following the needle puncture of the midgut. Circulating hemocytes quickly responded to the midgut wound by aggregating around the wound site. No cell division and apoptosis were observed around the wounds. However, DNA duplication was quickly enhanced surrounding the wound site. Eventually the needle-puncture wound was repaired. Because of the low production of antibacterial peptides and the capacity for repair, this method was further developed to inject small compounds that, due to volatility (e.g., ethanol) or odor, cannot be added as a food supplement. Thus, larval midgut injection may be a practical method for screening small molecules using insects as a model in the future.

## Results

### Needle-puncture wound in the insect larval midgut

**Figure 1:**
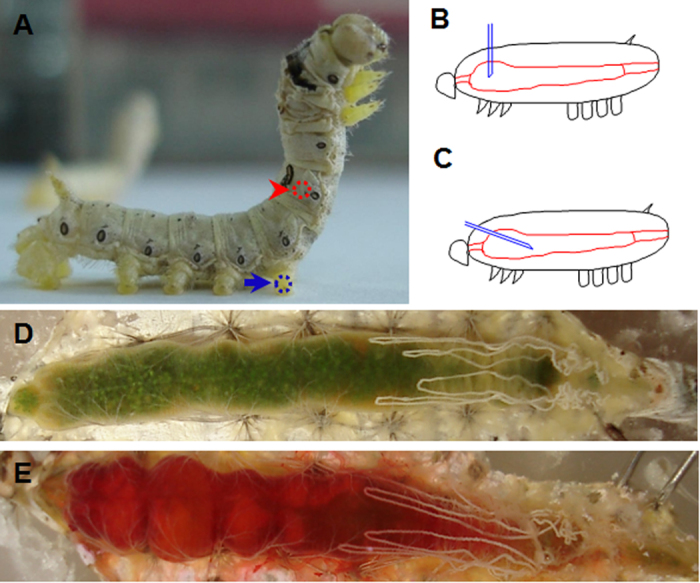
Insect midgut puncture and injection. (**A**) Position for making a needle puncture (arrowhead-indicated) in the midgut of a silkworm larva after the corresponding skin was sterilized. A control wound was made in one of the hind-legs as indicated by the arrow. (**B,C**) A diagram to show how needle puncture (**B**) and injection (**C**) were performed. A puncture was made by vertically probing the integument with half of the needle inside the body (**B**). If a solution was injected into the midgut, the needle was turned towards the hind of midgut (**C**). Morphology of a naïve midgut (**D**) or a midgut injected with 50 μl neutral red after 30 min (**E**). Less neutral red leaked with the hemocoel.

In the feeding larvae, wounds in the integument can be quickly repaired^11^. Currently, it is unknown whether and how a physical injury to the midgut is repaired in the feeding larva. To examine this, a sterilized needle (0.3 mm in diameter) was used to puncture the midgut of a silkworm larva at the arrowhead-indicated position (Fig. 1A). Half of the needle (0.7 mm in length) vertically penetrated through the integument (Fig. 1B). When it was necessary to inject solution into the midgut, the needle was positioned with a right-angle turn towards the back of the midgut (Fig. 1C). The midgut, as shown in Fig. 1D, was freshly dissected from a naïve larva. The midgut, as shown in Fig. 1E, was injected with 50 μl neutral red solution and dissected at 0.5 h post injection. Very little neutral red leaked from the midgut wound, which indicated that leaking after needle puncture was minor. Thus, the physical wound made by the needle, as shown in Fig. 1B,C, was suitable for the reported studies. As a control, a puncture was done in one of the hind-legs (arrow-point position in Fig. 1A).

**Figure 2:**
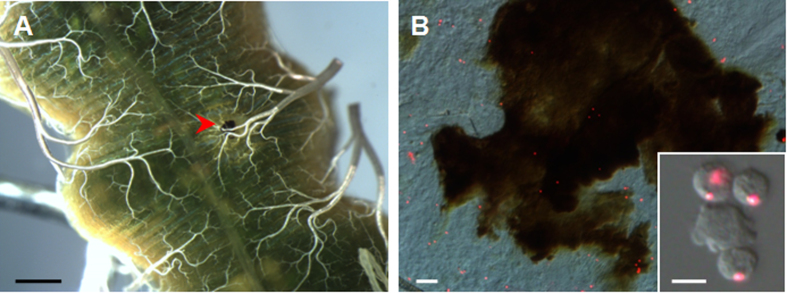
Circulating hemocytes are involved in midgut wound repair. (**A**) A melanized scab produced over the wound site. At 6 h after the wound was made, the midgut was dissected to show a melanized scab (arrowhead). (**B**) Hemocytes in the scab. Circulating hemocytes were pre-labeled via phagocytosis of injected fluorescent beads for at least 6 h as described^25^ and then a needle puncture was made. The inset is a picture to show hemocytes that had phagocytosed red fluorescent beads. The melanized scab was removed and pressed on a slide to observe fluorescent beads. The pictures were merged from those taken using a red filter and DIC optics. Bar: A, 1 mm; B, 20 μm; Inset in (**B**), 10 μm.

Initially, a wound was made in the midgut as shown in Fig. 1A. At 6 h, the punctured midgut was dissected and it was observed that the wound was melanized (Fig. 2A). In additional studies, circulating hemocytes with a phagocytosis function were pre-labeled by injecting red-fluorescent beads for at least 6 h as previously described^15^,^25^. Subsequently, a physical wound was made in the midgut and after another 6 h, the melanized material was stripped off the wound and the phagocytosed fluorescent beads were observed (Fig. 2B). These fluorescent beads were from circulating hemocytes as shown in the inset (Fig. 2B). These data demonstrate that a physical wound can be made in an insect midgut without serious leakage of midgut fluids and the wound is likely repaired via the involvement of circulating hemocytes.

### Gastrointestinal perforation does not induce sepsis in insects

**Figure 3:**
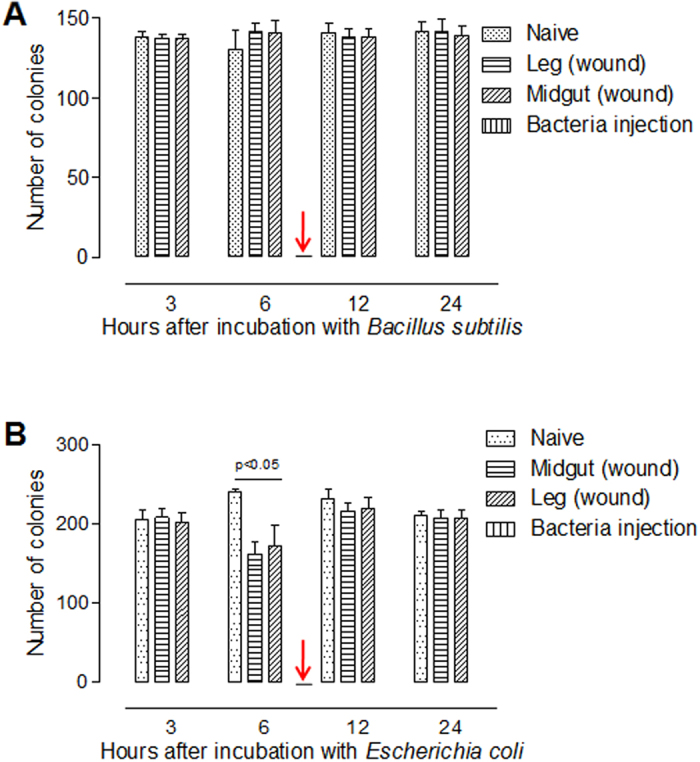
Inhibition of bacteria growth. Plasma was collected from silkworm larvae (V-3) receiving needle punctures in the midgut. Puncture made in the hind-leg was a control. Plasma from larvae receiving a bacteria immune challenge was a positive control (arrow-indicated). Plasmas from larvae receiving different treatments for the indicated time were collected and mixed with *B. subtilis* (**A**) or *E. coli* (**B**) separately for 90 min. The bacteria were cultured on a LB plate for counting colonies. Plasma of larvae from 6 h midgut punctures could partially inhibit the growth of *E. coli*. Each column represents the mean of measurements (±SE) from three biological replicates. An unpaired two-tailed *t*-test was performed to assess the significance of differences between groups unless otherwise stated.

The above needle-puncture wounds in the insect midguts are very similar to human gastrointestinal perforations that can cause sepsis^24^. Wounds in the midgut may induce similar disease or an immunity response in insects. Larvae received wounds to the midgut (including the integument) or the leg and these larvae, along with naïve ones, were bled at different times for the collection of plasma. After co-culture with bacteria, plasma from the midgut wound larvae or the hind-leg injured larvae did not inhibit the growth of *Bacillus subtilis* (Fig. 3A) while the plasmas from the larvae that received wounds to the midgut or hind-leg at 6 h did partially inhibit the growth of *Escherichia coli* (Fig. 3B). However, the plasma collected from larvae that had received a bacterial immune challenge killed both *B. subtilis* and *E. coli*. It is likely that the heated plasma inhibited bacterial growth due to the presence of antibacterial peptides^26^. The level of inhibition of *E. coli* by plasma from larvae that received wounds either to the midgut or leg was the same, indicating that midgut wounds did not induce an additional immune response as compared with wounds to the hind-leg. These data indicate that wounds made to the midgut did not induce a systemic immune response in insects.

### Repair of wounds to the midguts

When a larva received a wound to the midgut, a melanized scab was formed (Fig. 2A,B) and during this process, circulating hemocytes were involved. One hour after the puncture, hemocytes had already aggregated around the wound to form a melanized scab that was easily lost (Fig. S1A). Unlike an integument wound, the scab was located on the basal membrane of the midgut and protruded into the hemocoel. At 3 h, with many more hemocytes aggregated, the scab became larger (Fig. S1B). At this time point, the scab was strongly attached to the midgut surface, and it was difficult to remove. Subsequently, the scab grew larger 6–24 h post wounding (Fig. S1C–S1E). During the first 24 h, bubble-like materials were produced around the wounds on the side facing the midgut contents (Fig. S1A–S1E). At 48 h, the wound in the midgut was repaired, over which there was a large scab (Fig. S1F).

**Figure 4:**
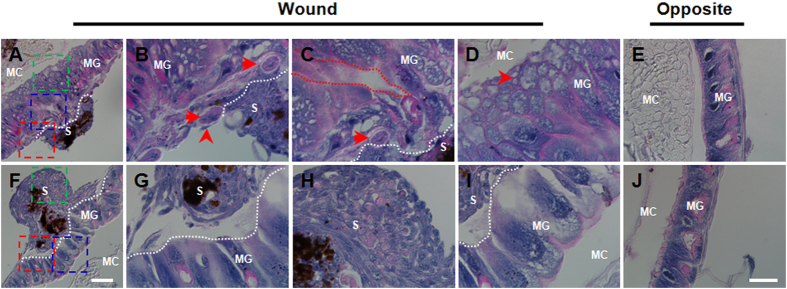
Close observation of the midgut wounds. At 6 h, the wound was undergoing repair (**A–D**). At 48 h, the wound was repaired as evidenced by the intact structure (**F–I**). The part of the midgut opposite the wound site at each time point is presented (**E,J**). At 6 h (**A**), the areas framed in red (**B**), blue (**C**) and green (**D**) lines were magnified for closer observation, respectively. At 48 h (**F**), the areas framed in red (**G**), blue (**H**) and green (**I**) lines were also magnified and the midgut and aggregated hemocytes in the scabs were compared. In (**B,C**), the arrows point to large muscles. The arrowhead points to a hemocyte connecting a muscle and the scab. The red line framed area in (**C**) indicates the puncture hole in the midgut. In (**C**), the arrowhead points to a bubble-like material. All white dotted lines separate the scab and midgut. MG, midgut; S, scab; MC, midgut contents. Bar: (**A,F**), 100 μm; (**B,C,D,G,H,I**), 25 μm.

When the wounds were closely observed and compared at 6 and 48 h separately (Fig. 4A,F), it was found that at 6 h, the muscles on the basal membrane became larger than those in other places (Fig. 4B). A single hemocyte was observed to attach to a muscle and its other end was connected to the scab (Fig. 4B). The contacting surface of the midgut and scab is visible (Fig. 4B) and a space that was likely left after the needle puncture is clear in appearance (Fig. 4C). On the inner surface facing the midgut contents, many bubble-like materials were noted (Fig. 4D). However, the section opposite the wound did not show this effect (Fig. 4E). By 48 h, the wound in the midgut was already repaired (Fig. 4F). Among the contact sites of midgut and scab, singular hemocytes formed a ligament-like material that had one end attached to the midgut and another end bound to the scab (Fig. 4G), showing that the scab was fixed to the wound via circulating hemocytes. In the scab, aggregated hemocytes produced melanin around the food and/or tissue debris (Fig. 4G,H). In the core of the scab, the initial hemocytes aggregated tightly. After scab formation, circulating hemocytes loosely attached to the scab (Fig. 4H). Two days later, the bubble-like material observed in the first 24 h disappeared, and the midgut was repaired as shown by the intact midgut cells (Fig. 4I). At this same time point, no change was observed in the section opposite of the wound (Fig. 4J). These data demonstrated that the needle puncture wound in the midgut was repaired within 48 h with circulating hemocytes involved.

### No apoptotic cell death around the wound site of midgut

**Figure 5:**
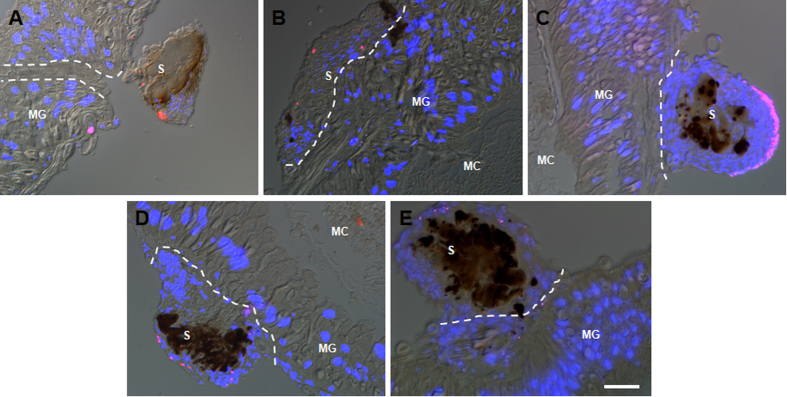
The puncture made in the midgut does not induce apoptosis in cells surrounding the wound. At 1 (**A**), 3 (**B**), 6 (**C**), 12 (**D**), and 24 h (**E**) after the punctures were made, the punctured midguts were dissected for the detection of apoptotic cells using the TUNEL method. Few midgut cells with apoptotic signals were detected around the wound. Some cells with apoptotic signals (red spots) were found in the scab. The white dotted lines separate the scabs and midguts. The pictures were merged from those taken using a red filter (TUNEL), blue filter (DAPI) and DIC optics. MG, midgut; S, scab; MC, midgut contents. Bar: 50 μm.

During the repair process, some bubble-like materials were produced around the midgut wounds by an unknown mechanism. This phenomenon raises the question as to whether there were some cells undergoing death surrounding the wounds. Therefore, cell apoptosis was assayed using the TUNEL method. No apoptotic cells were detected in the midgut wound at different times assayed (Fig. 5A–E). However, apoptotic signals were observed in the scab while it was forming (Fig. 5A). Although the scab was formed through hemocyte-mediated encapsulation, the number of apoptotic cells was not increased at 3 h (Fig. 5B). There was, however, one layer of cells surrounding the scab that were apoptotic at 6 h (Fig. 5C). Subsequently, there were only very limited apoptotic cells on the scab surfaces at 12 and 24 h, respectively (Fig. 5D,E) indicating that physical puncture to the midguts did not induce apoptosis around the wound.

### Needle puncture induces DNA duplication in cells surrounding the wound

**Figure 6:**
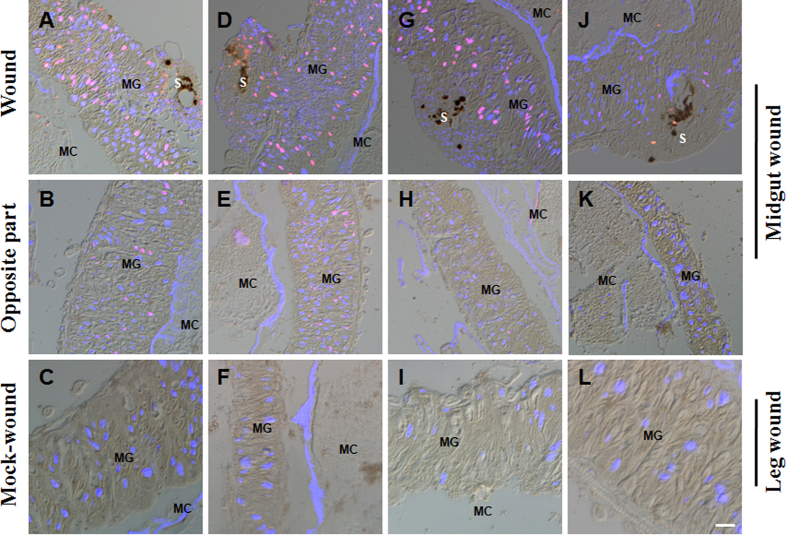
DNA duplication in cells surrounding the wound site. Silkworm larvae (48 h post ecdysis; day 3 of 5th feeding stage) that received wounds in the midguts or hind-legs were injected with BrdU to label DNA-duplicated cells at 3 (**A–C**), 6 (**D–F**), 24 (**G–I**), and 48 h (**J–L**), respectively. After the wounds were made, DNA duplication was assayed in cells surrounding the midgut wound site (**A,D,G,J**), the section opposite the midgut wound (**B,E,H,K**) and the region corresponding to the area above the midgut wound when punctures were made in one hind-leg (**C,F,I,L**). Many cells around the wound site had incorporated BrdU (red) according to the staining results. Some cells opposite the wound also incorporated BrdU. The wound in the hind-leg did not induce DNA duplication in the midgut cells. The pictures were merged from those taken using a red filter (BrdU), blue filter (DAPI) and DIC optics. MG, midgut; S, scab; MC, midgut contents. Bar: 50 μm.

Following the 4th larval ecdysis, there were many cells containing duplicated DNA on the first day of the 5th feeding stage (V-1) (Fig. S2A–S2D). As a baseline, we determined that no midgut cells incorporated BrdU at 36 h post 4th stage ecdysis (data not shown), and subsequently larvae on V-3 were selected for analyzing cell division and DNA duplication after needle puncture. In the silkworm wounded midgut, cell division was not observed since the phosphorylated histone-H3 (PH3), which is indicative of mitosis, was not detected (data not shown). When BrdU was injected immediately after needle puncture, many cells surrounding the wounds incorporated BrdU at 3 h post puncture (Fig. 6A). There were also some cells that incorporated BrdU opposite the wound in the midgut (Fig. 6B), which indicated that the influence of needle puncture on DNA duplication had spread. There were also many cells that incorporated BrdU around the wounds between 6 and 24 h (Fig. 6D,G). However, the cell number decreased compared to that at 3 h. In the opposite section of the wound, DNA duplication was observed at 6 h (Fig. 6E), but very few cells incorporated BrdU at 24 h (Fig. 6H). At 48 h when the midgut was repaired, there were several cells that had incorporated BrdU (Fig. 6J). No BrdU was incorporated into cells in the opposite section of the wound at that time point (Fig. 6H). When wounds were made in the hind-leg, no DNA duplication was observed in the midguts at all times assayed (Fig. 6C,F,I,L). These data demonstrate that needle puncture induced DNA duplication around the midgut wound.

### DNA duplication is essential for needle-puncture wound repair

**Figure 7:**
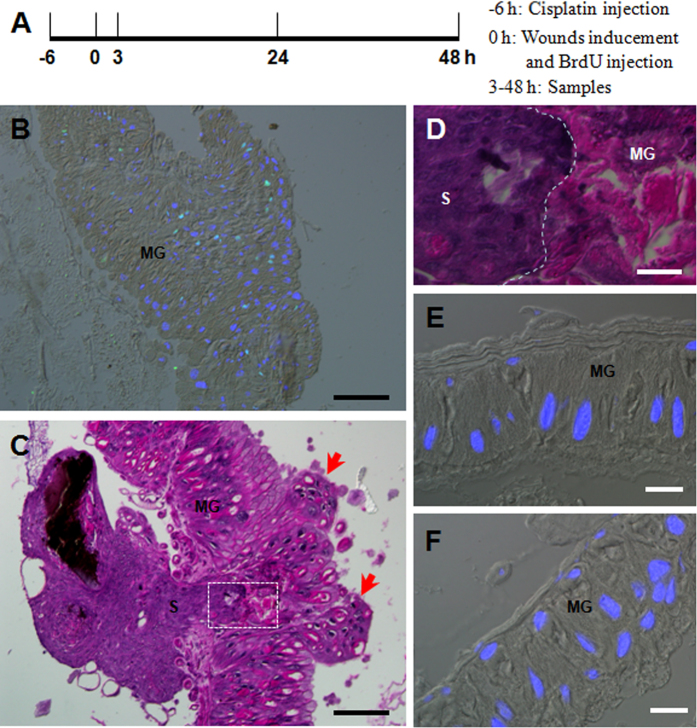
DNA duplication is essential to needle-puncture wound repair in the midguts. (**A**) A diagram to show cisplatin injection (-6 h), wound inducement and BrdU injection (0 h) in order to understand the importance of DNA duplication to the wound repair process. Samples were taken at 3 h to detect BrdU incorporation, and at 24 and 48 h for hematoxylin and eosin staining. (**B**) DNA duplication in cells surrounding the wound at 3 h since it was made (i.e., 9 h after cisplatin injection). The picture was merged from those taken using a red filter (BrdU), blue filter (DAPI) and DIC optics. (**C**) Morphology of the wound at 48 h (i.e., 54 h after cisplatin injection). The wound was not repaired and many cells were sloughed off the midgut (arrow-indicated). (**D**) A close-up observation of the neighboring sites of scab (S) and midgut (MG) as outlined in white in (**C**). The white dotted line indicates the neighboring surface between the hemocyte-aggregated plug-like scab and the midgut. (**E,F**) Injection of cisplatin did not induce cell apoptosis in midgut cells of naive larvae (**E**) or larvae that had received a needle-puncture 48 h previously (**F**). In (**F**), the injection area was outside of the needle-puncture wound region. The pictures were merged from those taken using a blue filter (DAPI) and DIC optics. Bar: (**B,C**), 100 μm; (**D–F**), 20 μm.

Cisplatin is one of the most potent antitumor agents that acts via cross-linking to DNA to form intra and inter strand adducts, thereby suppressing DNA synthesis^27^,^28^. A dose of 50 μg cisplatin was injected into each naive larva on the 1st day of the 5th feeding stage when DNA duplication can be observed in the midgut cells according to BrdU labeling (Fig. S2A–S2D). When cisplatin was injected, incorporation of BrdU, an indicator of DNA duplication, was obviously inhibited beginning at 6 h according to the assay (Fig. S2E-S2F). However, no BrdU-positive cells were detected in the midgut at 9 h (Fig. S2G). At approximately 24 h, some midgut cells were observed to incorporate BrdU again (Fig. S2H). These results indicate that cisplatin can inhibit DNA duplication in the midgut cells within a limited time period. At day 3 of the 5th larval feeding stage, no BrdU incorporation was observed unless a needle-puncture wound was made in the midgut (Fig. 6). In order to understand the importance of DNA duplication in midgut wound repair, cisplatin was injected 6 h prior to wound puncture of the midgut, and then BrdU injection was performed and midgut morphology was observed at different times as indicated (Fig. 7A). At 3 h post BrdU injection (9 h after cisplatin injection), cisplatin inhibited BrdU-incorporation (Compare Fig. 7B and Fig. 6A) although the inhibition was not complete. At 48 h after needle-puncture, the wounds were not repaired (Fig. 7C,D). The hemocyte-aggregated scab protruded into the wound like a plug, which is often observed in epidermal wounds^8^. Surrounding the midgut wound, some midgut cells were sloughed off the tissue (arrowed-indicated in Fig. 7C) and the scab inside the midgut was very clear (Fig. 7D). The injection of cisplatin did not induce cell apoptosis in the midguts of naive larvae or larvae that had received needle-puncture wounds (Fig. 7E,F). Obviously, the inhibition of DNA duplication blocked the repair of the needle-puncture wounds. These data demonstrate that DNA duplications is essential for midgut wound repair.

### Transport of small compounds into the insect midgut by injection

**Figure 8:**
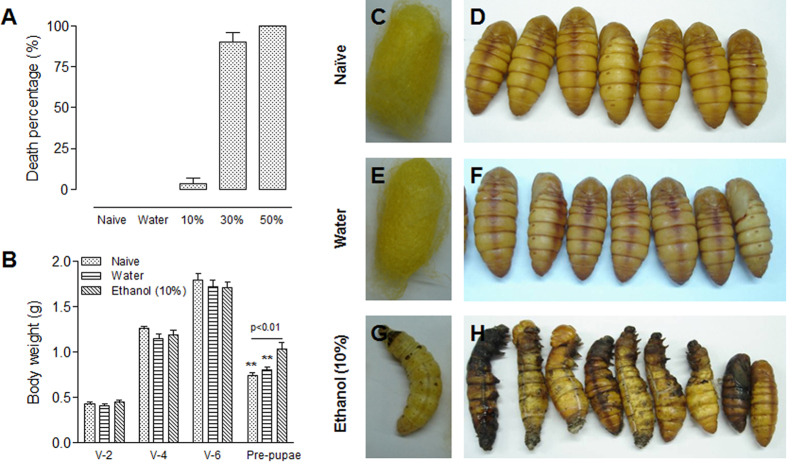
Concentrations of ethanol (10, 30 and 50%) injected into the midguts affect spinning and pupation. Ethanol solution at different concentrations was injected into the midguts of silkworm larvae (50 μl for each larva). (**A**) Percentage of death after ethanol injection. The number of dead larvae were counted at 24 h after injection. Each column represents the mean of measurements (±SE) from three biological replicates (at least 15 larvae for each treatment). (**B**) The lowest concentration of ethanol (10%) did not affect larval growth but did prevent spinning. There were no significant differences in body weight among different treatments during the feeding period. However, the weight of pre-pupae receiving the lowest concentration (10%) of ethanol injection during the feeding stage (V-2) was significantly higher than others. Each column represents the mean of measurements (±SE) from three biological replicates (at least 15 larvae for each treatment). (**C–H)** Morphology of cocoons and pupae receiving different treatments during the feeding stage (V-2) as indicated. (**C,D**) Naïve; (**E,F**) Water injection; (**G,H**) 10% ethanol injection. (**C,E,G**). Cocoons. Silkworms that received an ethanol injection into the midgut during the feeding stage (V-2) did not spin (**G**). (**D,F,H**) Pupae. Ethanol injection affected pupation. **p < 0.01.

Insects are an excellent model to screen small molecules for medicinal purposes^29^, but it is hard to feed each larva the same amount of molecules due to feeding difficulties. However, when an injection approach was tested and neutral red was injected into the midguts, very little leaked from the midgut after injection (Fig. 1E). Since the puncture wounds can be repaired quickly in the midgut and only weak immunity responses were induced in the whole larvae (Figs 3 and 4), it appeared to be feasible to inject small molecules into the midgut. In order to confirm this conclusion, we injected silkworm larvae (V-3) with the same volume of ethanol at different concentrations. At 3 and 6 h post injection, the midguts were dissected for comparison. When larvae were injected with a 10% ethanol solution, the midgut structures appeared almost the same as the naïve ones (Fig. S3A and S3C). At 6 h, it appeared that many eosin-stained materials filled the space between the midgut and the peritrophic membrane compared with the controls (Fig. S3B and S3D). However, when the insects were injected with a 30 or 50% ethanol solution, the midguts were seriously damaged (Fig. S3E- S3H). At 24 h after ethanol injection, over 90% of larvae were dead if the ethanol concentration was 30 or 50% (Fig. 8A). Less than 5% of larvae died if 10% ethanol was injected and no death was observed if water was injected. The body weights of living larvae after injection with 10% ethanol also were measured. There were no obvious differences among all treatments during the feeding stage (Fig. 8B); however, there were significant differences in pre-pupa body weight. Before pupation, the silkworms that received 10% ethanol injections had higher body weights as compared to the naive or water-injected controls. Naive silkworms and those injected with water spun normally and became pupae (Fig. 8C–F), but when silkworm larvae received 10% ethanol injections during the feeding stage, they could not spin even though no effect was observed on feeding and growth (Fig. 8B). Ultimately, the ethanol-injected silkworms did not become pupae (Fig. 8G,H). These data indicate the feasibility of injecting compounds into the insect midgut for screening purposes.

## Discussion

Wound repair is an important physiological event for invertebrate and vertebrate animals to maintain the integrity of their bodies. During the life cycle, each organism may receive various unexpected wounds caused by physical injuries, chemicals and/or pathogen invasion via the skin and/or gut. In order to maintain structural and physiological integrity, it is necessary to repair the wounds as quickly as possible. There are many similarities in wound repair between mammals and insects^23^,^30^. In *Drosophila*, wound repair in the integument and gut are different. There is no cell division surrounding an integument wound, and the JNK pathway drives the epidermal cell spreading and reepithelialization observed in such a wound^8^,^31^. In the midgut, apoptosis in the midgut enterocytes induces intestinal stem cell (ISC) proliferation and differentiation for wound repair^21^,^22^, which is controlled by the JAK/STAT pathway. Without those stem cells (ablated by genetically induced apoptosis), midgut repair cannot be completed^22^.

In humans, perforation occasionally occurs in the gastrointestinal tract which can lead to the leakage of gut contents and can then cause serious disease^24^. In these studies, using a needle, a physical puncture was made in the silkworm larval midgut, which is similar to a gastrointestinal perforation in humans. When silkworm larvae received a puncture wound to the midgut, it was not lethal. On the contrary, the physical wound to the midgut was repaired (Fig. 4). After a midgut wound, circulating hemocytes were found to aggregate around the wound to form a scab (Fig. 2B). This process happened very quickly, which is advantageous for wound repair. The physical wound in the midgut was repaired through DNA duplication since many midgut cells surrounding the wound incorporated BrdU within the first 6 h (Fig. 6). After that, the number of BrdU-labeled cells decreased. Therefore, in addition to involvement of circulating hemocytes, DNA duplication is important for the induction of wound repair in the midgut.

Insects are a good model to screen small compounds on a large-scale for medicinal purposes^29^. However, many chemical compounds may have an odor that precludes insects from directly ingesting the compound if mixed with food. In addition, it is impossible to control the amount of chemical ingested by insects. Consequently, in this study, we show that when neutral red was injected into the midgut, little leakage of the dye occurred within 30 min (Fig. 1E). This demonstrates that small compounds can be delivered into the insect midgut through injection for large scale screening purposes. Expanding upon this approach, we injected silkworm larvae with ethanol at different concentrations. Low concentrations of ethanol (10%) did not significantly damage the midgut (Fig. S3C-S3D), even though a few larvae may die from ethanol (Fig. 8A). Most larvae that survived the injection of 10% ethanol grew normally as compared with the naïve and water-injection larvae (Fig. 8B). Unfortunately, these larvae could not spin normally and most of them died before pupation (Fig. 8C–H). Insects share with mammals many similar biological pathways that regulate growth, development and immunity^23^,^30^. Therefore, using insects as a model, the injection of small compounds into insect midguts could be practical for screening small compounds for subsequent use as gut chemotherapeutics in humans.

## Methods

### Insect feeding

The silkworm *Bombyx mori* larvae (Nistari) were fed mulberry leaves at 25 °C with a 12-h photoperiod. Larvae on day 1–3 of the 5th larval stage (V-1 to V-3) were used for different purposes as described below. All methods were performed in accordance with approved guidelines and regulations. All experimental protocols were approved by the Institute of Plant Physiology and Ecology.

### Needle puncture to the midgut and injection

A disposable commercial syringe with a needle of 0.3 × 15 mm (Changqiang, China) was pressed against wax several times to plug the needle. This needle was used to vertically puncture the midgut or one of the hind-legs, which were sterilized with 70% ethanol beforehand. The silkworm larvae initially were anesthetized on ice for 20 min and then the selected body part was punctured with half of the needle (Fig. 1A,B). The silk gland was not damaged based upon observation after dissection. In order to inject neutral red (Sigma-Aldrich) or ethanol (Sinopharm Chemical Reagent Co., Ltd), the needle was not plugged with wax. After puncturing, the needle was vertically turned to the hind midgut (Fig. 1C). Approximately 50 μl solution was injected into each midgut. After puncturing or injecting, the silkworm larvae were placed on ice for another 20 min to stop bleeding and then the larvae were transferred to a clean paper for recovery. The silkworm larvae that received wound treatment were fed as usual.

### Immunity challenge and plasma collection

Silkworm larvae (V-3) that were injected with 5 × 10^6^ formalin-killed *Escherichia coli* cells 12 h previously were bled to obtain immunity-challenged plasma^15^. Silkworm larvae (V-3) that were punctured in the midgut or hind-legs were bled to obtain plasma at different times. All plasmas were then heated at 100 °C for 10 min. The plasma samples were then centrifuged at 10,000 g for 10 min at room temperature and the supernatants collected for bacterial growth inhibition studies.

### Larval plasma inhibition of microbial cell growth

A single colony of *E. coli* (Gram-negative) or *Bacillus subtilis* (Gram-positive) was cultured, collected and suspended in 0.85% autoclaved NaCl solution separately^4^ and then the optical density was measured at 600 nm (A_600_). Approximately 5 μl heated plasma was mixed with 45 μl suspended bacteria (*E. coli* 0.000003 OD, *B. subtilis* 0.00003 OD after serial dilution), respectively, and the above mixtures were incubated at 37 °C (at 200 rpm for 90 min). An additional 20 μl of the above mixture was used to separately streak a LB plate that was then maintained at 37 °C for 12 h for counting the colonies.

### Light microscopy

Tissue sectioning was performed as described^25^. Slides (5 μm) were stained using a mixture of hematoxylin and eosin solution to show morphological changes. All pictures were taken using a fluorescent microscope (Olympus BX51) with differential interference contrast (DIC) and the appropriate filter unless otherwise mentioned.

### Injection of cisplatin

Cisplatin (D109812; cis-Diammineplatinum(II) dichloride, Aladdin Industrial Corporation) was dissolved in water to make a 1.0 mg/ml solution. Each silkworm larva at the 5th feeding stage was injected with 50 μl Cisplatin solution based on the results of preliminary experiments. The control larvae were injected with 50 μl water. At the scheduled time points, larvae were injected with BrdU to label cells with duplicated DNA or were punctured in the midgut using a needle. Those midguts were then dissected and fixed for tissue sections.

### BrdU labeling and detection

Silkworm larvae that received wounds in the midgut or hind-leg at different times were weighed and anesthetized on ice and then injected with 5 μl BrdU Labeling Reagent (000103; Thermo Fisher Scientific Inc.) per gram body weight^32^. Three hours after injection, larvae were dissected to obtain the wounded midguts and the midguts fixed and sectioned as described above. A previously described method of BrdU labeling was followed to detect midgut cells that incorporated BrdU using mouse anti-BrdU monoclonal antibody (00–3900; Thermo Fisher Scientific Inc.) (1:200) as the primary antibody^25^. Alexa Fluor® 594 Goat Anti-Mouse IgG (H + L) Antibody (A-11032; Thermo Fisher Scientific Inc.) (1:1000) was the secondary antibody and DAPI (4′,6-diamidino-2-phenylindole) was used to counter-stain nuclei for fluorescent microscopy.

### *In situ* apoptosis detection: the TUNEL method

Midguts that had received wounds were fixed and sectioned at different times as described above. The *In situ* Cell Death Detection Kit, TMR red (Roche) was used to check for apoptotic cells following the manufacturer’s instructions. DAPI was used to counter-stain nuclei for fluorescent microscopy.

### Bead injection and detection

In the silkworm larvae, some hemocytes can phagocytose fluorescent beads^25^ and after phagocytosis, those hemocytes with fluorescence beads can be easily traced for different purposes^15^,^25^. To detect whether circulating hemocytes were involved in midgut wound repair, the insect larva was injected with approximately 9 × 10^6^ fluorescent microbeads [1.0 μm Φ, Red (580/605); Molecular Probes] as previously described^25^. At 6 h post fluorescent microbead injection, needle punctures were made in the midguts. The resulting scabs from the midgut wounds were then removed for microscopy after another 6 h.

## Additional Information

**How to cite this article**: Huang, W. *et al*. DNA duplication is essential for the repair of gastrointestinal perforation in the insect midgut. *Sci. Rep*. **6**, 19142; doi: 10.1038/srep19142 (2016).

## Supplementary Material

Supplementary Information
