# Supplementary material for: DNA duplication is essential for the repair of gastrointestinal perforation in the insect midgut
Source: Sci Rep. 2016 Jan 12;6:19142. doi: 10.1038/srep19142 (PMC4709577; doi:10.1038/srep19142)
Supplement: Supplementary Information [file srep19142-s1.doc]

**Supplementary information:**

**DNA duplication is essential for the repair of gastrointestinal perforation in the insect midgut**

Wuren Huang^1^, Jie Zhang^1^, Bing Yang^1^, Brenda T. Beerntsen^2^, Hongsheng Song^3*^, Erjun Ling^1*^

^1^Key Laboratory of Insect Developmental and Evolutionary Biology, Institute of Plant Physiology and Ecology, Shanghai Institutes for Biological Sciences, Chinese Academy of Sciences, Shanghai 200032, China

^2^Veterinary Pathobiology, University of Missouri, Columbia, MO 65211, USA

^3^ College of Life Sciences, Shanghai University, Shanghai 200444, China

**Running title:** DNA duplication in the wound midgut

***To whom correspondence may be addressed.**

Hongsheng Song: hssong@staff.shu.edu.cn; Erjun Ling: [ejling@sibs.ac.cn](mailto:ejling@sibs.ac.cn)

**Keywords:** Gastrointestinal perforation, midgut, DNA duplication

**Supplemental figures and legends**


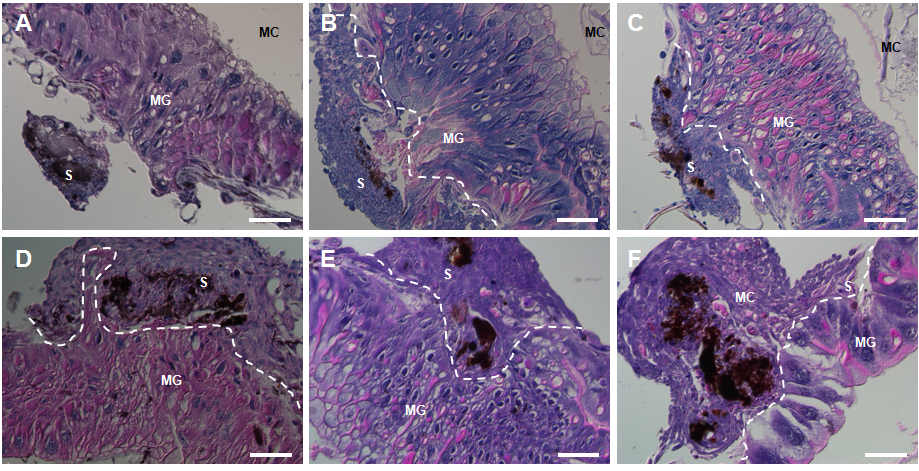


**Figure S1.** Morphological changes of the needle punctured midguts. After puncture with a needle in the midguts at the same position, the midguts were dissected for tissue (the neighboring segment outside of wound) sectioning at 1 (A), 3 (B), 6 (C), 12 (D), 24 (E), and 48 h (F), respectively. The dashed white line separates the scab (S) and midgut (MG). The wounded midguts were repaired within 2 days. MG, midgut; MC, midgut contents. Bar: 50 μm.


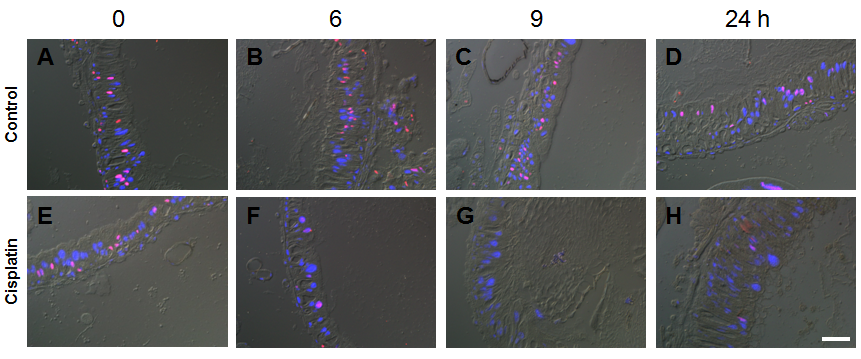


**Figure S2.** Injection of cisplatin inhibit DNA duplication. Cisplatin (50 μl, 1 mg/ml) was injected into larvae on day 1 of 5th feeding stage (V-1). The control larvae were injected with the same volume of water (50 μl). At each indicated time, the midguts were sampled to detect BrdU through immuno-staining. DNA duplication was inhibited among 6-9 h. At 24 h, some cells incorporated BrdU again. The pictures were merged from those taken using a red filter (BrdU), blue filter (DAPI) and DIC optics. Bar: 100 μm.


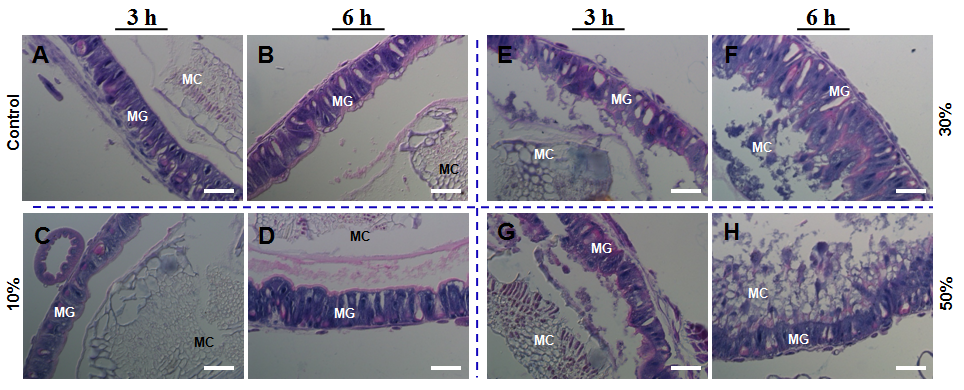


**Figure S3.** Injection of high concentrations of ethanol into the midguts causes serious damage. As described in Fig.1, different concentrations of ethanol solution were injected into the midguts of silkworm larvae. At 3 h and 6 h, those midguts were dissected for tissue sectioning, respectively. After comparison with control ones injected with water (A, B), ethanol at low concentration (10%, 50 μl) did not significantly damage the midgut (C, D). However, high concentrations of ethanol (30-50%, 50 μl) seriously damaged the midgut (E-H). Bar: 50 μm.
